# Mechanisms, Biomarkers, and Treatment Approaches for Diabetic Kidney Disease: Current Insights and Future Perspectives

**DOI:** 10.3390/jcm14030727

**Published:** 2025-01-23

**Authors:** Jean Paule Joumaa, Angela Raffoul, Charbel Sarkis, Elizabeth Chatrieh, Sally Zaidan, Philippe Attieh, Frederic Harb, Sami Azar, Hilda E. Ghadieh

**Affiliations:** Department of Biomedical Sciences, Faculty of Medicine and Medical Sciences, University of Balamand, Al-Koura, Tripoli P.O. Box 100, Lebanon; jeanpaule.joumaa@std.balamand.edu.lb (J.P.J.); angela.raffoul@std.balamand.edu.lb (A.R.); charbel.sarkis@std.balamand.edu.lb (C.S.); elizabeth.chatrieh@std.balamand.edu.lb (E.C.); sally.zaidan@std.balamand.edu.lb (S.Z.); philippe.attieh@std.balamand.edu.lb (P.A.); frederic.harb@balamand.edu.lb (F.H.); sami.azar@balamand.edu.lb (S.A.)

**Keywords:** Diabetic Kidney Disease, diabetic nephropathy, endstage renal disease, SGLT-2 inhibitors, inerenone, hyperglycemia, albuminuria, proteinuria

## Abstract

Diabetic Kidney Disease (DKD) is the leading cause of end-stage renal disease (ESRD) worldwide. Among individuals with type 1 diabetes mellitus (T1DM), 30–40% are at risk of developing DKD. This review focuses on the mechanistic processes, available and emerging biomarkers for diagnosing, monitoring, and preventing DKD, as well as treatment options targeted at DKD patients. A literature search was conducted on PubMed and Scopus using specific keywords. Inclusion and exclusion criteria were applied to select the articles used for this review. The literature highlights various mechanisms involved in the progression of DKD to more severe stages. Additionally, several biomarkers have been identified, which aid in diagnosing and monitoring the disease. Furthermore, numerous treatment approaches are being explored to address the underlying causes of DKD. Advanced research is exploring new medications to aid in DKD remission; sodium-glucose cotransport (SGLT2) inhibitors and finerenone, in particular, are gaining attention for their novel renoprotective effects. DKD is a major complication of diabetes, marked by complex and multifactorial mechanisms. Thus, understanding these processes is essential for developing targeted therapies to potentially reverse DKD progression. Biomarkers show promise for early diagnosis and monitoring of disease progression, while current treatment strategies underscore the importance of a multifaceted approach.

## 1. Introduction

Diabetic kidney disease (DKD), also referred to as diabetic nephropathy (DN), has been attributed as the leading most common cause of end-stage renal disease (ESRD) [1]. Research has shown that around 30–40% of patients living with type 1 diabetes mellitus (T1DM) develop DKD, and approximately 50% can progress to ESRD [2]. DKD has been associated with genes and phenotypes that are affected by genetic factors and signal transduction pathways [2]. Statistics demonstrate that, in 2021, the prevalence of DKD in men was comparable to that in women and increased steadily with age [3,4].

The pathogenesis of DKD is linked to several interactions corresponding to metabolic, inflammatory, hemodynamic, and multifactorial causes [2]. Mechanistically, DKD stems from impaired glucose regulation, leading to glucotoxicity, which is poorly tolerated by the kidneys. Furthermore, oxidative stress and mitochondrial dysfunction, along with renal hypoxia, have been implicated in the progression and worsening of DKD. Additional contributing factors include podocyte injury, renal endothelial dysfunction, and proteinuria. Thus, DKD can be divided into two unique phenotypes: the classical albuminuric, and the non-albuminuric phenotype. The former is associated with signs of diabetic glomerulopathy on histology; while the latter presents with atherosclerosis, atypical vasculature, and fibrosis with intact glomerular structure [5]. Scientists project that the number of renal replacement treatments will rise from 2.819 million to 4.35 million by 2035 due to DKD compromising patient care and decreasing their quality of life [5]. Currently, the lack of effective diagnostic methods with sufficient specificity and sensitivity often leads to delayed detection of DKD until irreversible damage has occurred [4]. Moreover, dealing with DKD entails dealing with several challenges, as conventional techniques, such as renin angiotensin-aldosterone system (RAAS) blockage and glycemic monitoring, do not reverse or stop the condition from progressing [6]. Diagnostic biomarkers are diverse, encompassing laboratory tests, imaging techniques, and molecular markers that offer insights into disease progression and status. Treatment options are also varied, providing physicians with a range of choices that differ in efficacy. This review concentrates on the mechanistic process through which DKD progresses, the available and novel biomarkers used in the diagnosis, monitoring, and prevention of DKD, and the treatment options targeted to help DKD patients.

## 2. Methods and Results

The search strategy for this review involved using the key terms “Diabetic Kidney Disease” OR “Diabetic Nephropathy” combined with Boolean operators to include “Mechanism*” OR “Pathogenesis”, “Biomarker*” OR “Marker*” OR “Assay*”, and “Treatment*” OR “Therapeutic*” OR “Therap*” OR “Cure”. Inclusion criteria were limited to full-text meta-analyses, reviews, and systematic reviews published between 2014 and 2024, focusing on human subjects and written in English. Exclusion criteria included preprints, articles and studies prior to 2014, randomized controlled trials (RCTs), clinical trials, books, and book chapters. The initial database search retrieved 871 articles, with 221 duplicates removed, leaving 641 records for screening. Subsequent exclusions based on title irrelevance (258), abstract incompatibility (165), and further detailed analysis for irrelevance (118) and redundancy (39) resulted in a final selection of 51 reports included in this review.

**Figure 1:**
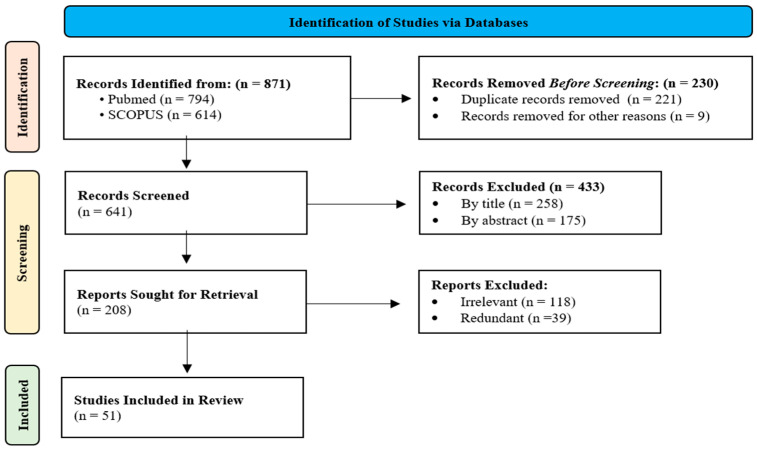
Flow diagram illustrating the selection process of the included studies.

Figure 1 shows the flow diagram of the selection process of the included studies.

## 3. Discussion

The mechanisms contributing to DKD are interconnected, and the development and progression of this disease involves a complex interplay among various factors. These mechanisms have been associated with specific biomarkers, which have been used to diagnose and assess kidney function in diabetes, but some have low specificity and sensitivity for DKD. This has led to research into novel biomarkers for earlier and more accurate detection of kidney damage in DKD. Furthermore, several treatments for DKD have been developed over the years. However, as the leading cause of ESRD and a major contributor to mortality and morbidity in diabetes patients, treatment alone is insufficient. Preventing the development and progression of DKD is crucial. Recently, new studies have focused on novel treatments and prevention strategies.

### 3.1. Primary and Initiating Mechanisms of DKD

#### 3.1.1. Pathological Mechanism

Hyperglycemia

DKD is caused by several multifactorial pathophysiologic mechanisms, such as structural, hemodynamic, metabolic, inflammatory, and fibrotic pathways, which are mainly the result of hyperglycemia [1,6,7,8,9,10,11,12,13,14]. The pathogenesis can involve hyperglycemia induced overactive RAAS, inflammation, oxidative stress, tubular damage, and many mediators that overlap and interact together [1,6,7,8,9,10,12,13,15]. Moreover, albuminuria and glomerular filtration rate (GFR) are the main markers of DKD and the development of macroalbuminuria may lead to ESRD [8,16,17,18].

Advanced Glycation End Products (AGEs)

AGEs form when proteins react non-enzymatically with glucose, causing permanent changes in protein structure, which can impair normal function. They cross-link proteins, disrupting their activity, and signal through receptor for advanced glycation end products (RAGE). By interacting with multiple cell surface receptors, such as toll-like receptors (TLRs), G-protein-coupled receptors (GPCRs), scavenger receptors (SRs), and pattern recognition receptors (PRRs), AGEs trigger cellular damage through downstream effects [6]. In diabetes, AGEs activate key signaling pathways through RAGE, leading to the chronic activation of inflammatory transcription factors, which worsens tissue injury, this can include p38, mitogen-activated protein kinase (MAPK), stress-activated protein kinase/jun N-terminal kinase (SAPK/JNK), and the Jjanus kinase/signal Ttransducer and activator of transcription (JAK/STAT) pathway [6].

Renin–Angiotensin–Aldosterone System (RAAS)

Renin, produced by juxtaglomerular cells near the afferent arteriole, initiates RAAS, causing greater vasoconstriction in the efferent arteriole. Angiotensin-converting enzyme 2 (ACE2) converts AngII into angiotensin, which dilates the glomerular afferent arterioles [7,19]. AngII activates angiotensin II type 1 and type 2 (AT1 and AT2) receptors: AT1 increases efferent arteriole resistance, contributing to hyperfiltration, while AT2 promotes renal vasodilation and prostaglandin release. High AngII levels accelerate renal damage by increasing calcium influx into podocytes, stimulating proinflammatory cytokines, matrix Mmetalloproteinase-9 (MMP-9), and transforming growth factor-β (TGF-β), and activating macrophages. This also enhances aldosterone secretion, upregulating profibrotic factors and promoting kidney fibrosis [5,8]. Additionally, AngII induces vascular constriction, oxidative stress via nicotinamide adenine inucleotide Pphosphate (NADPH) oxidase, and increased myocardial contractility, all contributing to DKD pathophysiology [17].

#### 3.1.2. Biomarkers

Advanced Glycation End Products (AGEs)

Patients with diabetes have elevated AGE levels, which accumulate in the glomeruli and tubules in DN [4]. Increased AGE production is driven by oxidative stress and hyperglycemia, and high AGE levels correlate with reduced renal function [4,10]. The upregulation of sodium-glucose cotransporter 2 (SGLT2) cotransporters increases glucose reabsorption, putting proximal epithelial cells at risk of hypoxic injury and AGE formation [20]. In patients with ESRD, AGE levels are twice as high as in those without renal disease [4]. AGEs bind to RAGEs, triggering proinflammatory pathways and oxidative stress [10,15]. Pentosidine, an AGE, is elevated in microalbuminuria and early GFR decline and may predict diabetic retinopathy, cardiovascular disease, and mortality, making it an early biomarker for DKD [20].

ß2 microglobulin (ß2M)

ß2M is a protein localized on the cell surface of nucleated cells as part of MHC class I. It is shed by cells into the blood and is normally filtered and almost completely reabsorbed by the kidneys. Serum ß2M is strongly correlated with serum Cystatin C (CysC) and creatinine (Cr) and can be used for assessment of GFR as it is negatively correlated with it [21], and positively correlated with urinary creatinine-to-albumin ratio (uACR) [22]. A study in African DM patients showed that increased urinary ß2M occurs at least as early as microalbuminuria and was associated with tubulopathy [23]. In another study, T1DM patients were found to have increased expression of ß2M in the urinary sediment with initial or overt DN compared to T1DM patients without DN. Moreover, serum levels are associated with increased risk of mortality in patients with chronic kidney disease (CKD) and diabetes [22]. However, more studies must be carried out to determine whether ß2M levels rise before the onset of microalbuminuria in DN patients to ensure that it may be used as a reliable early biomarker for kidney damage.

#### 3.1.3. Treatments and Therapies

Glucose Control

Intensive blood glucose control in T1D can correct glomerular hyperfiltration in the short term and, when sustained for 8 to 60 months, can reduce the progression of nephropathy. It also lowers albuminuria and the risk of reduced GFR by 50% [8]. Medications, like glucagon-like peptide-1 receptor agonists (GLP-1 RA) and dipeptidyl peptidase 4 (DPP-4) inhibitors, slow albuminuria, while SGLT-2 inhibitors reduce renal disease progression and the need for dialysis. These drugs are recommended as second-line therapy for patients who do not reach their hemoglobin A1C (HbA1C) target with lifestyle changes and metformin alone [17,24].

SGLT2 Inhibitors

SGLT2 inhibitors block sodium and glucose reabsorption in the kidneys, reducing blood glucose levels, with a low risk of hypoglycemia. SGLT2 inhibitors exert renoprotective effects by correcting glomerular hyperfiltration, improving kidney oxygenation, and reducing mitochondrial damage, which leads to anti-inflammatory and antifibrotic effects through decreased oxidative stress and enhanced tubule-glomerular feedback [25,26,27,28]. SGLT2 inhibitors, such as empagliflozin, dapagliflozin, and tofogliflozin, offer cardiovascular and renoprotective benefits in T2D through multiple mechanisms. Empagliflozin improves kidney function by correcting glomerular hyperfiltration, enhancing kidney oxygenation, and reducing oxidative stress [29]. Dapagliflozin ameliorates DN by improving hyperglycemia, reducing β-cell damage and albuminuria, and suppressing inflammation and oxidative stress in both diabetic mice and cells [30]. Tofogliflozin preserves pancreatic β-cell function and prevents kidney dysfunction in *db/db* mice, suggesting that long-term use may help prevent DN progression [31].

While the primary and initiating mechanisms of DKD set the stage for its development, oxidative stress and its associated pathways play a crucial role in the progression and worsening of the condition.

### 3.2. Oxidative Stress and Associated Mechanisms in DKD

#### 3.2.1. Pathological Mechanism

Oxidative Stress

In DKD, chronic hyperglycemia drives increased reactive oxygen species (ROS) production and oxidative stress through mechanisms such as AGE formation, the polyol pathway, and protein kinase C (PKC) activation [7,15,18,32]. This is exacerbated by reduced endothelial nitric oxide synthase (eNOS) levels [7]. Additionally, lactic acidosis and hypoxia contribute to mitochondrial dysfunction and fibrosis, as observed in mouse models of DKD [15,32]. In the tubulointerstitial regions, ROS activation induces podocyte apoptosis, inflammatory factor release, and renal fibrosis signaling, ultimately impairing glomerular filtration function and consistently elevating TGF-β1 levels, promoting fibrosis [15,18,32].

Mitochondrial dysfunction

The association between DKD and mitochondrial dysfunction stems from hyperglycemia-driven mechanisms, where increased ROS production from electron transport chain overload leads to DNA damage and reduced glyceraldehyde-3-phosphate dehydrogenase (GAPDH) activity [8]. Alternatively, reduced mitochondrial superoxide then stimulates inflammatory processes via vascular dysfunction and the nuclear factor kappa-light-chain-enhancer of activated B cells (NF-κB) [8,9,17]. Eventually, mitochondrial dysfunction leads to activation of apoptosis and cell death [12]. Several mitochondrial disorders affecting the kidney typically manifest as tubulopathies or cystic renal diseases, with glomerular involvement being rare [9]. Moreover, it was found that several genes related to mitochondria were methylated differently in patients with ESRD [2].

Renal Hypoxia

Vascular remodeling, microvascular dysfunction, impaired oxygen diffusion due to extracellular matrix (ECM) accumulation, anemia, and mitochondrial abnormalities are key factors contributing to kidney hypoxia [33]. Consequently, glomerular and vascular lesions reduce oxygen supply, leading to renal medulla hypoxia and damage from free radicals in advanced DKD. The main mediators of metabolic hypoxia are hypoxia-inducible factors (HIFs), including HIF-1, HIF-2, and HIF-3. In hypoxia, HIF degradation is inhibited, leading to HIF-α accumulation. HIF-α then dimerizes with HIF-β to form functional HIFs, which translocate to the nucleus and activate gene transcription. However, hyperglycemia can destabilize HIFs, promoting tissue fibrosis. As a result, higher blood sugar levels correlate with increased tissue damage [8]. DKD is characterized by an increase in activation of HIF-1α, alongside HIF-2α suppression, which can contribute to glomerular and renal tubular injury.

Glucotoxicity in DKD

In DM, oxidative stress is a consequence of glucotoxicity and lipotoxicity, which play a part in the pathogenesis of β-cell dysfunction [34]. The hyperglycemic state in DM leads to oxidative stress, which further inhibits insulin secretion. Furthermore, a reduction in glutathione (GSH), an increase in oxidized glutathione (GSSG) (calculated as GSH:GSSG ratio), and a decrease in non-enzymatic antioxidants are perceived. This leads to the formation of a viscous cycle of hyperglycemia causing increased oxidative stress that further decreases insulin secretion, which in turn results in hyperglycemia [34]. Additionally, in the mitochondria, oxygen is used to obtain energy, and for the action of microsomal oxidases and cytosolic pro-oxidant enzymes. The generation and accumulation of ROS lead to oxidative stress, hyperglycemia, and increased insulin resistance [35].

Understanding the pathological mechanisms of oxidative stress in DKD provides valuable insights into potential biomarkers, which can aid in early detection, monitoring, and assessment of disease progression.

#### 3.2.2. Biomarkers

Vascular Endothelial Growth Factor (VEGF) and Receptor (VEGFR)

VEGF is a growth factor involved in angiogenesis, vascular permeability, and kidney development, essential for forming the glomerular filtration membrane [36]. VEGF-A, primarily expressed by podocytes, maintains endothelial function at low levels, while VEGFR-2 is expressed by endothelial cells [36,37]. Increased VEGF and VEGFR2 expression in early DKD may be triggered by inflammation and hypoxia to promote repair [36,38]. However, VEGF overexpression can disrupt the glomerular filtration barrier and promote fibrosis [38]. Reduced VEGF-A in advanced DKD results from podocyte loss [36,37]. Thus, monitoring VEGF levels can track disease progression, but its dual role in repair and fibrosis must be considered in management.

Advanced Glycation End Products (AGE)

Diabetes patients have higher levels of AGEs, which accumulate in the DN’s glomeruli and tubules [4]. Increased AGE synthesis is caused by oxidative stress and hyperglycemia, and high AGE levels are associated with impaired renal function [4,10]. Upregulation of SGLT2 cotransporters promotes glucose reabsorption, putting proximal epithelial cells at risk of hypoxia damage and AGE production [20]. Patients with ESRD have twice as high AGE levels as individuals without renal disease [4]. AGEs attach to RAGEs, activating proinflammatory pathways and causing oxidative stress [10,15]. Pentosidine, an AGE, is higher in microalbuminuria, and early GFR decrease and may predict diabetic retinopathy, cardiovascular disease, and death, making it a potential early biomarker for DKD [20].

Soluble Klotho (α-Klotho)

α-Klotho is a transmembrane protein that consists of domains K1 and K2, which can be cleaved by MMP. A soluble form of Klotho, generated from the cleavage of its domains, is the main form found in the circulation and is also present in blood, urine and cerebrospinal fluid [39]. Klotho is heavily implicated in the aging process through its role in phosphate homeostasis, insulin signaling, the integrity of blood vessels and in other complex signaling pathways, and its levels tend to decrease with age [39]. Kidneys are major contributors to the levels of circulating Klotho: it is mainly expressed in the podocytes, proximal and distal tubules, and moves through the basolateral, intracellular and apical spaces and eventually into the urinary lumen [40]. Klotho levels decrease in both T1DM and T2DM: both plasma and urine levels decrease in early DN, as low levels correlate with impairment of renal function and the onset of albuminuria [41] and with progressive decrease in these levels with DKD progression [40]. However, soluble Klotho levels must be studied for further evidence that the changes in these levels are exaggerated by the aging process or other disease processes and taking into consideration that statins and angiotensin receptor blockers (ARBs) may interfere with Klotho levels [41].

The identification and validation of biomarkers not only enhance our understanding of DKD but also pave the way for the development of targeted treatments and therapies aimed at mitigating disease progression.

#### 3.2.3. Treatments and Therapies

Mineralocorticoid Antagonists

Finerenone, a selective nonsteroidal mineralocorticoid receptor antagonist, has shown promising results in the management of DKD, particularly in T2D, where it has been proven to slow the progression of kidney damage. By blocking the mineralocorticoid receptor, finerenone reduces inflammation and fibrosis in the kidneys, thereby protecting against the decline in GFR and albuminuria. The FIDELIO–DKD [42] study demonstrated that finerenone significantly improved renal outcomes, including a reduction in the risk of kidney failure and a sustained decline of at least 40% in estimated glomerular filtration rate (eGFR) in patients with T2D and CKD, as well as a decrease in uACR [43]. Finerenone has demonstrated cardiovascular benefits in addition to its renal protective effects, providing a dual advantage [42,44]. However, a key safety concern with finerenone is the risk of hyperkalemia which requires careful monitoring, especially in patients with impaired renal function [45]. While finerenone has primarily been studied in T2D patients [43], ongoing research may confirm its efficacy in patients with T1D and DKD; see the ongoing phase 3 FINE-ONE study [46]. However, given its pleiotropic effects, it is possible that finerenone may offer additional benefits in terms of β-cell preservation in diabetic patients. Currently, the direct effects of finerenone on β-cell function are not fully understood, and more research is needed [47].

Dietary Intervention

Patients with DKD should follow the American Diabetes Association’s (ADA) recommendation of 0.8 g of protein per kg per day to slow GFR decline and progression to ESRD. A Mediterranean diet rich in whole-grain carbohydrates, fiber, fruits, vegetables, omega-3 and omega-9 fats, and less than 2300 mg of sodium per day, can help control hypertension and improve outcomes. Additionally, patients with altered phosphorus, vitamin D, or potassium levels may require further dietary adjustments [8,14,24].

While oxidative stress plays a central role in the pathogenesis of DKD, its interplay with hemodynamic factors further exacerbates kidney injury and disease progression, highlighting the complex nature of the disease.

### 3.3. Hemodynamic Impacts

#### 3.3.1. Pathological Mechanism

Hemodynamic Changes

Elevated blood glucose levels stimulate increased SGLT2 activity, resulting in enhanced reabsorption in the proximal tubules. This leads to a downregulation in tubuloglomerular feedback stimuli because of low sodium delivery to the macula densa [5,6,8,9]. Accordingly, afferent arteriolar dilation occurs alongside elevated angiotensin II (AngII) levels in the efferent arteriole, causing vasoconstriction [5,20]. This ultimately leads to an increase in GFR [8,9] and the development of glomerular hypertension, which is the major cause of diabetic glomerulopathy. To prevent glomerular damage, it is essential to control glomerular capillary pressure in addition to reducing systemic blood pressure [8]. Moreover, the increase in GFR is the first step in the pathogenesis of DKD, as it could lead to albuminuria, and kidney failure or ESRD [6]. Renal vasoconstriction reduces blood flow at the glomerular filtration barrier, potentially inducing metabolites, like lactate and L-serine. These metabolites contribute to podocyte damage, accelerated cell aging, increased inflammation, tubular fibrosis, and endothelial dysfunction [18].

Glomerular Hypertension

Vascular remodeling and narrowing from increased resistance often precede a rise in blood pressure, creating a cycle where endothelial dysfunction further accelerates vascular remodeling. Ultimately, systemic hypertension, along with hyperglycemia and hyperinsulinemia, leads to medial and intimal thickening of the afferent arterioles, further promoting glomerular hypertension [48]. This leads to dysfunction in the contraction and dilation of the afferent arterioles, impairing the regulation of glomerular pressure. In diabetes, afferent arteriole dysfunction allows glomerular pressure to exceed 70 mmHg while, in healthy individuals, glomerular pressure is maintained at 50 mmHg despite systemic hypertension [48].

The pathological mechanisms underlying DKD provide a foundation for identifying specific biomarkers, which can serve as critical tools for early diagnosis, monitoring disease progression, and assessing therapeutic responses.

#### 3.3.2. Biomarkers

Albuminuria and Proteinuria

Microalbuminuria and macroalbuminuria, caused by structural changes in the GBM, are key features of DN and are commonly used to define the condition. However, albuminuria is not a sole biomarker for DKD, as it can be influenced by factors like exercise, heart failure, and urinary tract infections [13]. Proteinuria may not appear before renal function declines, suggesting early tubulointerstitial involvement in kidney damage. Therefore, additional biomarkers are needed for early DKD diagnosis [19].

Creatinine and Estimated Glomerular Filtration Rate (eGFR)

Creatinine is used to estimate GFR, and eGFR is a key parameter for assessing kidney function and diagnosing DKD [19]. While albuminuria and a decline in eGFR are clinical features of DKD, they are not specific to the condition [19]. Tubular damage, another aspect of DKD, is not reflected by eGFR but by urinary tubular markers. Therefore, eGFR alone is insufficient for assessing DKD severity and progression, and additional biomarkers are necessary [17,19].

Fibroblast Growth Factor-23 (FGF-23)

FGF-23, produced by bone osteocytes and osteoblasts, regulates phosphate levels by decreasing sodium-phosphate cotransporter expression in the proximal tubules and reducing intestinal absorption through decreased Vitamin D levels [49,50]. Elevated FGF-23 levels are found in T2DM patients, albuminuria, cardiovascular disease (CVD), and mortality, though some studies report no link between diabetes and blood FGF-23 levels [49]. Insulin reduces FGF-23 so, in T1DM, low insulin leads to high levels while, in T2DM, insulin resistance may reduce them [50]. Inflammation, common in T2DM, can elevate FGF-23. AGEs and SGLT inhibitors also increase FGF-23 levels. FGF-23 rises early in DN before phosphate levels increase, and its levels significantly increase as the disease progresses, making it a potential early biomarker for DKD detection and monitoring [50,51].

Cystatin C (CysC)

CysC, a cysteine protease, is a better GFR indicator than creatinine, with higher sensitivity and specificity in both type I and II diabetes, even before albuminuria appears [4]. Unlike creatinine, CysC is not affected by muscle mass or diet, making it a more reliable GFR estimator. However, CysC can still be influenced by factors like inflammation and fat mass [7]. Urinary Cystatin C (uCysC), normally absent in urine, increases with tubular injury or dysfunction. uCysC is a useful tubular marker for diagnosing DKD and predicting albuminuria progression in T2DM patients [20].

#### 3.3.3. Treatments and Therapies

Blood Pressure Control

ACE inhibitors and ARBs effectively delay and reduce DKD progression. ACE inhibitors also lower the risk of new-onset microalbuminuria or macroalbuminuria and reduce mortality in diabetic patients, regardless of hypertension [26]. However, combining ACE inhibitors with ARBs offers no added benefits and increases the risk of hyperkalemia, hypotension, and renal failure. While RAS inhibitors (ACE-I and ARB) lower proteinuria and slow GFR decline, they cannot fully prevent progression to ESRD [26].

Statins

DKD alters lipid metabolism, increasing LDL-cholesterol levels and the risk of adverse atherosclerotic cardiovascular outcomes. While statins do not significantly impact DKD progression, they reduce cardiac events and mortality in non-dialysis-dependent renal disease patients [52]. Since many statins are renally metabolized, doses should be adjusted for patients with significantly low eGFR, except for atorvastatin, which requires no adjustment. Trials on statin use in hemodialysis patients show mixed results with limited relative benefit [14,32].

Bone Health

Patients with DKD, especially those with T1DM, are at higher risk of fragility fractures. This is linked to disturbances in bone-regulating factors, including relative hypoparathyroidism and changes in hormones, like FGF-23, sclerostin, and klotho, leading to bone disease. Supporting bone health may involve reducing dietary phosphorus, especially from processed foods with additives like trisodium phosphate, and/or using phosphate binders. Additionally, healthcare provider-recommended vitamin D supplements, calcimimetics, or calcium supplements can be beneficial [53,54].

### 3.4. Cellular and Structural Injuries

#### 3.4.1. Pathological Mechanism

Podocyte Injury

Podocytes line the external surface of the glomerular basement membrane (GBM) and form a barrier preventing urinary protein loss [1,16]. When these cells lose their adhesive properties, they detach from the GBM, resulting in proteinuria [1,16]. The loss of podocytes triggers the proliferation of mesangial cells, which subsequently leads to fibrosis [16]. Hyperglycemia triggers podocyte apoptosis through pathways involving ROS, renin-angiotensin system (RAS), mammalian target of rapamycin (mTOR), and transforming growth factor-beta/SMAD (TGF-β/SMAD) signaling pathway, contributing to podocyte loss and albuminuria in DKD [1]. Moreover, because of their limited regenerative capacity, podocyte loss in DKD is often irreversible, exacerbating glomerular injury and proteinuria. Furthermore, activation of the JAK–STAT pathway in podocytes, and mitochondrial dysfunction is linked to increased albuminuria and decreased podocyte numbers [1].

ECM Accumulation and GBM Thickening

DKD is characterized by the accumulation of ECM, leading to thickening of the GBM, which impairs its filtration function and contributes to fibrosis [6]. Protein kinase C (PKC) activation, particularly PKC-α and PKC-β under high glucose conditions, stimulates the production of key ECM components, such as fibronectin, laminin, and collagen, in renal glomerular mesangial cells, exacerbating matrix accumulation and kidney fibrosis [6]. TGF-β1 further drives ECM accumulation, worsening GBM thickening and promoting fibrotic changes in the kidney [6]. GBM thickening, a hallmark change in diabetic patients, alters its structure and collagen composition, increasing permeability and proteinuria [13,32]. While the exact role of GBM thickening in DKD progression remains unclear, it is both a consequence of and a contributor to declining kidney function, ultimately worsening outcomes and increasing mortality in advanced DKD [6,13,32].

Epithelial–Mesenchymal Transition (EMT)

EMT is a process in which kidney tubular epithelial cells lose their epithelial characteristics and adopt a mesenchymal, myofibroblast-like phenotype, contributing to fibrosis and kidney injury in DKD [6]. TGF-β promotes EMT by converting epithelial and endothelial cells into mesenchymal cells, which increases pro-fibrotic activities, leading to kidney tissue scarring [6]. Additionally, AngII induces ROS, promoting EMT, which causes tubular cell injury and contributes to fibrosis through the accumulation of myofibroblasts [12].

Proteinuria

Proteinuria is strongly linked to mesangial denaturation or glomerular necrosis, which can progress to kidney failure [48,55]. When excess protein reaches the renal tubules, it is usually reabsorbed by the tubular epithelium. However, a variety of intracellular pathways, involving the endoplasmic reticulum and lysozymes, become overwhelmed with increased proteinuria [55]. As a result, tubular cells produce cytokines that promote inflammation and attract inflammatory cells, such as macrophages [55]. Albumin binds free fatty acids and long-chain acyl-CoA metabolites, inducing changes in tubular cell function that may lead to lipo-apoptosis [55]. Moreover, stressed renal tubular cells produce profibrotic mediators, such as TGF-β1 [55]. Renal tubular cells migrate to the interstitium, where, along with interstitial fibroblasts, they contribute to ECM production and fibrosis [55].

The cellular and structural injuries associated with DKD trigger a cascade of molecular events, leading to the release of biomarkers that reflect the extent of tissue damage and can be used to assess disease progression and response to treatment.

#### 3.4.2. Biomarkers

Kidney Injury Molecule-1 (KIM-1)

KIM-1 is a transmembrane glycoprotein expressed in renal proximal tubule cells, with serum levels rising in response to tubular damage [19]. It is linked to eGFR decline and DKD progression, even in early stages with normo- and microalbuminuria [7,19]. Urinary KIM-1 (uKIM-1) is a more specific and sensitive biomarker than urine albumin, with levels increasing from normo- to micro- to macroalbuminuria inT1DM and T2DM. Both serum and uKIM-1 can serve as early biomarkers for DKD progression [20].

Transforming growth factor β (TGF-β)

TGF-β is a growth factor involved in renal fibrosis and DKD development through increased ECM deposition and glomerular hypertrophy [20]. Its activity is influenced by circular RNAs and MMPs, both linked to DKD [56,57]. TGF-β levels are higher in T2DM patients with macroalbuminuria, making it a useful biomarker for predicting disease progression [20].

Connective Tissue Growth Factor (CTGF)

CTGF, a member of the CCN protein family, mediates intercellular signaling and is linked to kidney fibrosis through interactions with TGFβ and other proteins, increasing collagen expression [5,58]. In DN, urinary CTGF levels rise due to both reduced reabsorption and increased local production [59]. In patients with T2DM and DKD, elevated CTGF expression in kidney biopsies correlates with proteinuria, serum creatinine, and fibrosis [58]. Increased serum and urinary CTGF are independent predictors of ESRD, making CTGF a potential biomarker for monitoring DN progression [58]. Table 1 summarizes the DKD biomarkers, their functions, and their roles in DKD progression.

Neutrophil gelatinase-associated lipocalin (NGAL)

NGAL (lipocalin-2) is a protein synthesized by neutrophils and injured nephrons, making it a useful biomarker for DKD [19]. It is released specifically upon nephron injury, with levels in urine and serum predicting albuminuria before it appears, thus serving as an early marker for nephron damage in diabetes [19,20]. NGAL can also help distinguish between diabetic and non-DKD through the urinary NGAL-to-creatinine ratio with high specificity [20].

Osteopontin (OPN)

OPN was originally known for its role as a major sialoprotein of bone involved in mineralization, remodeling and cellular adhesions, but is now known not only for its presence in bone but also for its expression in various cells of the immune system, epithelial and endothelial cells, adipocytes, and in fetal and mature renal tissue [60]. A modification through cleavage of the full-length OPN (OPN-FL) can yield a cleaved form, i.e., the N-half form. The kidneys contain a large amount of OPN content, and its major sources are the loop of Henle and the distal nephron, but glomeruli and all segments of the tubules increase OPN expression up to 18-fold with kidney damage [61]. OPN appears to contribute to the pathogenesis of DN through alteration of podocyte signaling and motility which leads to microalbuminuria, and through macrophage build-up in the interstitium, which contributes to tubulointerstitial damage [61]. In DN patients, full OPN levels were shown to be higher than in healthy subjects [60]. Moreover, in T1DM patients, OPN concentrations were higher with microalbuminuria and could predict its onset [60]. Furthermore, in patients with T2DM, OPN levels were found to be correlated with DN progression, with an inverse correlation between OPN and eGFR [60]. Full OPN levels also had a strong association with DN severity, while the N-half did not [60]. Therefore, plasma full length OPN may serve as a reliable marker for the onset of DKD and its development.

Hepcidin

Hepcidin is a liver-produced hormone that regulates iron homeostasis by inhibiting iron absorption in the gut and its release from macrophages and liver stores. Elevated hepcidin levels reduce iron availability, while low hepcidin levels increase iron absorption. Despite its well-established role in iron regulation, hepcidin has been identified as a biomarker predictive of clinical outcomes and mortality progression in DKD [62,63]. The literature reports that DKD and CKD patients are found to have increased levels of hepcidin [64]. Furthermore, a study investigating the predictive value of Hepcidin-25, the active isoform of the hormone hepcidin, for mortality and disease progression in DKD, found an independent association between hepcidin-25 levels, endogenous erythropoietin (EPO) levels, and kidney function impairment, after adjusting for inflammatory markers [62]. Hepcidin plays a crucial role in the pathogenesis of renal anaemia in CKD, and its elevated levels not only contribute to functional iron deficiency and resistance to erythropoiesis-stimulating agents but also may serve as a valuable biomarker for both renal anaemia management and cardiovascular disease prognosis [65]. In addition, elevated hepcidin levels are linked to better erythropoiesis-stimulating agent (ESA) response and may play a significant role in CKD-related complications, such as bacterial infections and atherosclerosis, independent of ESA hypo-responsiveness [66].

#### 3.4.3. Treatments and Therapies

Medications to Reduce Proteinuria

ACE inhibitors and ARBs are first-line antihypertensive therapies for patients with proteinuria due to their blood pressure-independent antiproteinuric effects. If hypertension persists, adding a diuretic is recommended. To further reduce proteinuria, combining an ACE inhibitor with an ARB or using other drugs known to decrease protein excretion, such as non-dihydropyridine calcium channel blocker or aldosterone receptor blockers, may be effective [67,68].

Smoking Cessation

Smoking accelerates kidney deterioration in diabetic patients, worsening microalbuminuria and proteinuria, shortening the time between their onset and diabetes, and hastening the progression of DN to ESRD. Smokers experience a faster GFR decline compared to non-smokers. Conversely, quitting smoking significantly improves albuminuria, with nephropathy progression rates lower in non-smokers (11%) compared to smokers (53%) and those who quit smoking (33%) [5,8,26].

### 3.5. Inflammation and Immunological Cycles

#### 3.5.1. Pathological Mechanism

Immune System Involvement

Proinflammatory cytokines (such as TNF-α, Interleukin-1 (IL-1)), chemokines, adhesion molecules, and damage-associated molecular patterns (DAMPs) [5,6,7,13,18,69] are released in response to cell injury caused by hyperglycemia, driven by factors such as vascular and glomerular lesions, obstructive sleep apnea, and intermittent hypoxia [7,8]. Damage primarily begins with macrophage accumulation, which generates ROS, and through mechanisms driven by hyperglycemia-induced AGEs [5,8,69]. This leads to destruction of glomerular podocytes, protein dysfunction, and metabolism impairment of the ECM [8]. Persistent hyperglycemia increases the formation of immune complexes, amplifying the innate immune response through complement activation and immunoglobulin Fcγ receptors (FcγRs). This process involves the renal mononuclear phagocyte (MNP) system, comprising macrophages and dendritic cells, which contributes to DKD progression [8,10,12]. These cells release free radicals, MMPs, proinflammatory cytokines, hydrogen peroxide and chemokines [5,6,10,13,18,69]. In fact, TNF-α has been shown to be related to the ESRD in diabetes [5,7].

The role of autophagy

Autophagy is crucial for kidney function regulation under normal and pathological conditions. In diabetes, autophagy is negatively regulated by three nutrient-sensing pathways: mTORC1 activation, AMP-activated protein kinase (AMPK), and Sirtuin 1 (SIRT1) inhibition [12,70]. mTORC1 activation in podocytes leads to DKD characteristics, such as mesangial expansion, GBM thickening, podocyte loss, and proteinuria in diabetic mice [12,70]. AMPK activation, via Ca^2+^/CaM-dependent protein kinase kinase β (CaMKKβ) and TGF-β-activated kinase 1 (TAK1) pathways, promotes autophagy in kidney cells [70]. Declining SIRT1 activity with age suppresses autophagy, contributing to renal phenotypes and DN [70]. Podocytes rely on basal autophagy, and mice lacking the autophagy protein 5 (ATG5) gene in podocytes show increased albuminuria, podocyte loss, and glomerulosclerosis [70]. In mesangial cells, TGF-β1-induced autophagy through the TAK1 and Phosphoinositide 3-Kinase/Protein Kinase B (PI3K/AKT) pathways enhances cell survival. Mitophagy and elevated ROS levels in the renal cortex also contribute to adenosine triphosphate (ATP) depletion in diabetic kidneys [12,70].

#### 3.5.2. Biomarkers

Interleukin-6 (IL-6)

IL-6 is a cytokine that regulates inflammation and is elevated in DKD patients [10]. Serum and urinary IL-6 levels increase early, before albuminuria, and progress from micro- to macroalbuminuria in both T1DM and T2DM [10,20]. Measuring urinary IL-6, along with other inflammatory markers, can help assess glomerular damage and disease progression [71].

Monocyte chemoattractant protein 1 (MCP-1)

MCP-1 (CCL2) is a pro-inflammatory chemokine secreted by mononuclear and mesenchymal cells, including kidney resident cells. It is found in kidney biopsies from DKD patients, with increased urinary MCP-1 levels linked to renal inflammation, glomerular and tubular damage, and fibrosis [20,69]. Glomerular injuries in DKD increase MCP-1 due to mesangial matrix deposition and protein leakage [72]. In normotensive, normoalbuminuric T1DM patients, urinary MCP-1 levels correlate with early nephropathy changes [20,71]. High urinary MCP-1 is associated with albuminuria progression and early GFR decline, though it does not always predict the onset or progression of albuminuria [20,69].

TNF-α and TNF-α receptors (TNFR) system

TNF-α is a proinflammatory cytokine that interacts with TNFR1 and TNFR2 receptors [72]. While TNFR2 is not typically present in kidney tissue, it is found in DKD patients [72]. Hyperglycemia in diabetes increases TNF-α release, primarily from monocytes, macrophages, and renal cells. TNF-α can be toxic to glomerular and mesangial cells, contributing to kidney damage [7,72]. Studies show high TNF-α levels in patients with advanced kidney disease and proteinuria. Urinary TNF-α, rather than serum levels, may be more closely linked to disease progression [7,20,72]. Elevated TNFR levels are strong predictors of macroalbuminuria and kidney disease progression in both T1DM and T2DM, suggesting that urinary TNF-α and circulating TNFR levels may serve as biomarkers for DKD progression [10,72].

Podocyte membrane protein urokinase receptor (uPAR) and its circulating form (suPAR)

Soluble urokinase plasminogen activator receptor is the soluble form of uPAR, which is a membrane-bound protein that acts as a urokinase (a serine protease) receptor. uPAR expression has been noted in kidney podocytes and tubular cells, from which it can be released as a soluble molecule through cleavage [73]. Healthy patients with high levels of suPAR were found to be more likely to have an annual decrease of up to 4.2 mL/min with remarkably elevated suPAR levels [73]. In healthy people who are at higher risk of DM, high serum suPAR levels predict microalbuminuria early on, and are associated with increasing proteinuria and decreasing GFR in DM patients [74]. Furthermore, patients with DN being treated with RAS blockage showed decreased urinary suPAR levels with no difference in serum levels [74]. This indicates that serum and urinary suPAR levels can be used as biomarkers for the prediction of onset of kidney damage and follow up treatment through serum suPAR.

#### 3.5.3. Treatments and Therapies

Kidney Replacement Therapy (KRT) and Kidney transplantation (KT)

Dialysis therapy: There is no specific eGFR value to start dialysis; personalized care is essential. Primary vascular access should not rely on tunneled catheters, as establishing vascular access in patients with limited life expectancy can significantly impact their quality of life. For KRT, it is unclear if the initial choice of modality, hemodialysis (HD) or peritoneal dialysis (PD), significantly affects outcomes, metabolic profile, diabetes complications, or technique survival. Patients should receive unbiased information to make informed decisions [75].

Renal Transplant: KT is the preferred treatment for diabetic patients with Kidney Failure with Replacement Therapy (KFRT), offering improved quality of life and long-term survival. Preemptive transplantation, done before dialysis, enhances survival in both diabetic and non-diabetic patients, emphasizing the need for early nephrologist referral and timely evaluation. Living donor KT further reduces waiting times, increasing the likelihood of avoiding dialysis [76].

### 3.6. Emerging and Regulatory Factors

#### 3.6.1. Pathological Mechanism

Genetic and Epigenetic Factors and Predisposition

Susceptibility to DKD is linked to genetic factors and familial clustering [6]. The treatment of DKD and the administration of appropriate therapies, guided by newly emerging evidence on the molecular pathogenesis of this disease, are crucial for maintaining kidney function in patients [77]. Epigenetic modifications, including DNA methylation, histone post-translational modifications (PTMs), and non-coding RNAs, can be influenced by hyperglycemia, hypoxia, inflammation, and cytokines. Epigenetic regulation is influenced by the patient’s environment and can be inherited across generations by offspring [77]. For example, apoptosis of glomerular cells in early DKD involves the gene UNC13B [2,6,8]. miRNA-192, which is abundant in kidneys, increases in early DKD, upregulating genes like Collagen Type II Alpha Chain-1 (Col2α1) and Collagen Type IV Alpha Chain-1 (Col4α1) in mesangial cells [5,6,8]. Changes in histone acetylation regulate microRNAs (miRNAs), while histone methylation affects TGF-β1-mediated ECM expansion. Nuclear Factor Erythroid 2-Related Factor 2 (Nrf2) offers protection in diabetes by inhibiting TGF-β1 and reducing ECM production. Genome-wide association studies (GWAS) have identified more than 250 genetic loci significantly impacting kidney function. Recently, several epigenetic alterations have been recognized as crucial for understanding the link between the genome and patients’ susceptibility to developing DKD [77]. Long noncoding RNAs (lncRNAs) also play crucial roles in DKD pathophysiology, influencing mesangial cells, podocytes, and ROS production [5,78]. A study investigating the long non-coding RNA metastasis-associated lung adenocarcinoma transcript 1 (MALAT1) in high glucose-induced glomerular endothelial dysfunction demonstrated epigenetic inhibition of *Klotho* transcription through the recruitment of the methyltransferase G9a [77]. These findings suggest that glomerular cells may interact with each other, contributing to the pathogenesis of DKD. Epigenetics opens the door to a new array of treatment modalities and interventions. Understanding this technology in order to precisely edit epigenetic elements is considered a promising approach for the future.

Metabolic Disturbances and Gut Microbiota Imbalance

**Figure 2:**
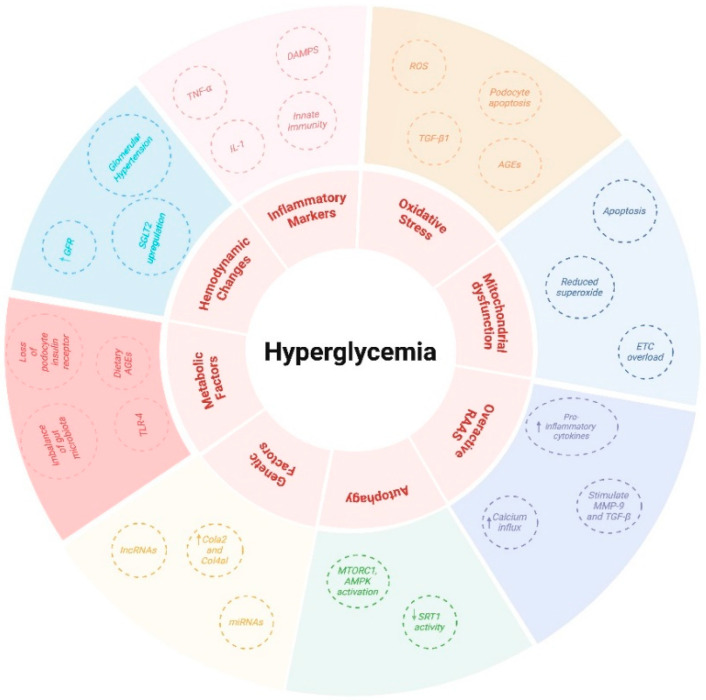
Mechanisms Underlying DKD Induced by Hyperglycemia. Upward arrows indicate an increase, while downward arrows indicate a decrease. Created in https://BioRender.com.

DKD results from metabolic disturbances, including hyperglycemia, hyperlipidemia, and insulin resistance. These factors increase the production of vasoactive mediators, like AGEs and ROS [5]. Mice lacking podocyte insulin receptors develop severe albuminuria and glomerulosclerosis [79]. Imbalances in gut microbiota, particularly species like *Prevotella copri* and *Bacteroides vulgatus*, are linked to insulin resistance and influence metabolism [79]. Increased gram-negative bacteria, such as *Klebsiella oxytoca*, promote endotoxemia, triggering inflammation via TLR-4, damaging podocytes, and causing albuminuria [5]. Gut microbiota dysregulation also leads to podocyte insulin resistance by inhibiting AMPK-α activity [79]. Butyrate may mitigate insulin resistance and renal dysfunction by enhancing AMPK phosphorylation and GLP-1 secretion [79]. AGEs from diet worsen glomerular changes, interact with the gut microbiota, and disrupt the intestinal barrier, facilitating inflammation and systemic kidney damage [5]. Figure 2 elucidates the mechanisms underlying DKD induced by hyperglycemia.

Urinary Exosomes

Exosomes play a role in the pathogenesis of DKD by transporting kidney-damaging contents between cells. For instance, exosomes derived from glomerular endothelial cells (GECs) can carry TGF-β1, which induces EMT and causes damage to podocytes [80]. Exosomes derived from glomerular mesangial cells (GMCs) treated with high glucose have been shown to induce podocyte injury via the TGF-β1-PI3K/AKT pathway. Additionally, these exosomes promote GMC proliferation and fibrosis through elevated levels of circ_DLGAP4, which sponges miR-143, as well as increased amounts of RAS components [80]. Macrophages exposed to high glucose concentrations released exosomes that enhanced macrophage activity, leading to the secretion of inflammatory and fibrotic cytokines, including TNF-α, TGF-β1, and IL-1β, as well as increased inflammasome activity mediated by miR-21-5p [81]. However, exosomes can also carry protective factors: exosomes derived from mesenchymal stromal cells have demonstrated anti-fibrotic, anti-inflammatory, and anti-apoptotic effects on tubular epithelial cells, along with anti-proliferative effects on podocytes, proving to be reno-protective in DKD [80].

#### 3.6.2. Biomarkers

MicroRNAs

MicroRNAs are short RNA sequences that regulate gene expression and can influence gene activation [82]. In DN, certain microRNAs (e.g., miR-21, miR-29a, miR-29b, miR-29c) are elevated, while others (e.g., miR-126, miR-192) are reduced. MicroRNAs contribute to DKD progression through inflammation, oxidative stress, insulin resistance, and glomerular injury [82]. Changes in microRNA expression correlate with albuminuria development [20]. For example, miRNA-155 increases TGF-β and TNF-α expression, promoting glomerular damage [82]. miRNA-27a overexpression activates the Wnt/β-catenin pathway, leading to podocyte dysfunction [3]. miRNA-21 correlates with renal fibrosis and function decline, suggesting that microRNAs could serve as early biomarkers for DKD [82].

Circular RNAs (circRNAs)

circRNAs are stable RNA molecules with closed ring structures, resistant to degradation. They influence gene expression by binding to miRNAs, RNA-binding proteins, and transcription enzymes, and some can encode proteins. CircRNAs play a role in the pathophysiology of DN, with their levels affecting disease progression [56]. For example, decreased CircRNA_010383 increases proteinuria and renal fibrosis, while elevated Circ_0125310 and Circ_DLGAP4 promote mesangial cell proliferation and fibrosis. CircHOMER1 increases oxidative stress, inflammation, and ECM deposition. Thus, circRNAs could serve as biomarkers for monitoring DN [56].

Urinary Exosomes

Urinary exosomes can be obtained from the urine through various non-invasive isolation processes. Since plasma exosomes do not normally pass through the glomerular filtration barrier, the exosomes obtained in the urine tend to originate mainly from cells of the urogenital system, reflecting the state of the kidneys [80]. Podocytes treated with high glucose exhibited elevated WT1 expression in vitro. In patients with DKD, high levels of WT1 mRNA in exosomes were associated with a rapid decline in eGFR [83]. Nephrin, a molecule essential for normal podocyte structure and cell signaling, was decreased in patients with elevated levels of urinary exosomal miRNA-22, reflecting the severity of the disease in DKD patients [83]. Elf3 serves as a highly specific marker for DKD as it is only found in the exosomes of patients with diabetes mellitus patients who have developed DKD [80]. Urinary exosomal miR-133b-3p, miR-342-3p, and miR-30a-5p may be elevated in diabetic patients with normoalbuminuria and increase significantly with the progression to micro- or macro-albuminuria in patients with T2DM, potentially serving as early indicators of DN [80]. miR-21-5p and miR-23b-3p were elevated in patients with T2DM and were linked to altered renal function, renal sclerosis and fibrosis [81], and miR-451 was correlated with renal failure [80]. Some exosomal miRNAs, such as miR-29c-5p and miR-15b-5p, may be decreased and are correlated with the progression of DKD [80]. There is a large number of existing exosomal biomarkers, as well as those yet to be explored, that correlate with various stages of DN progression. Some of these biomarkers exhibit high specificity, and this heterogeneity in exosomes can be leveraged as a tool to track the progression of DKD.

#### 3.6.3. Treatments and Therapies

Gut Microbiota Supplements

Dietary nutrient intake impacts gut microbiota and its link to CKD progression. Variation in protein intake affects the gut microbiota and DKD progression: plant-based, low-protein diets delay kidney replacement therapy by modulating RAS, reducing proteinuria, and improving insulin resistance. Whole-grain diets enhance gut bacterial diversity, alleviating kidney injury by reducing inflammation and immune signaling pathways [79]. Probiotics improve lipid profiles and anthropometric indices in DKD patients. Statins, primarily used for cardiovascular risk reduction, are recommended for diabetic patients with albuminuria but do not directly influence DKD progression. They lower cardiac event risks and mortality in non-dialysis-dependent DKD patients, with dose adjustments necessary for reduced eGFR, except for atorvastatin [5,79,84].

Hyperglycemia may trigger several mechanisms, including hemodynamic changes, inflammatory factors, oxidative stress, mitochondrial dyfunction, overactive RAAS, autophagy dysregulation, genetic factors, and metabolic disturbances. Hyperglycemia can lead to increased GFR, glomerular hypertension, and SGLT2 upregulation, contributing to hemodynamic changes. It may also upregulate inflammatory factors, such as TNF-α, IL-1, and DAMPs, and activate innate immunity. Additionally, oxidative stress results from increased blood glucose, involving ROS, TGF-β1, podocyte apoptosis, and AGEs. Mitochondrial dysfunction, associated with electron transport chain overload and reduced superoxide, further contributes to apoptosis. Overactivation of the RAAS stimulates proinflammatory cytokines, MMP-9, TGF-β, and calcium influx. Autophagy is triggered by mTORC1 and AMPK activation, along with declining SIRT1 activity. Furthermore, genetic and metabolic factors, including lncRNAs and miRNAs, contribute to hyperglycemia-induced mechanisms, with upregulation of Col2α1 and Col4α1. Metabolic factors include gut microbiota imbalance, podocyte insulin receptor deletion, increased TLR-4, and dietary AGEs.

Treatment with Exosomes

Exosomes are stable intercellular transporters, whose roles depend on the characteristics of their cargo, offering significant therapeutic potential if the cargo consists of renoprotective components. For example, urine-derived stem cell exosomes rich in miR-16-5p may inhibit VEGF-A in podocytes exposed to high glucose, playing a protective role against podocyte injury and inflammation, and could help reduce inflammatory responses [83]. Another example is that of exosomes extracted from human urine-derived stem cells, which have the potential to inhibit podocyte apoptosis, promote vascular regeneration, and enhance cell survival through caspase-3 inhibition in diabetic animal models [81]. Treatments that interfere with the intercellular transport of exosomes may also be beneficial, as demonstrated by Tongxinluo treatment in DKD, which inhibits renal fibrosis by blocking TGF-β1 transport via exosomes [80]. This suggests that exosomes can be utilized in various ways to delay kidney disease progression, either by loading them with protective factors or by preventing the transport of exosomes containing damaging factors.

## 4. Future Directions

DKD impacts and is influenced by various physiological systems and organs, including the cardiovascular, endocrine, gastrointestinal, and musculoskeletal systems. However, some areas of exploration remain underrepresented in the literature. Therefore, future research should further investigate the relationship between DKD and other physiological systems.

DKD and the Cardiovascular System

Patients with DKD are at an elevated cardiovascular risk, which worsens as DN progresses. The leading causes of death in these patients are cardiovascular events, and they also face a high risk of developing kidney failure and cardiovascular death [72,75,85]. Furthermore, hypertension is by itself a risk factor for DKD [86]. For individuals with cardiovascular disease or kidney failure, it is advised to aim for a blood pressure target of 130/80 mmHg [86]. Hypertension, especially when not properly managed, plays a crucial role in the progression of DKD. In T1DM, the transition from microalbuminuria to macroalbuminuria can contribute to hypertension. In T2DM, hypertension is often a characteristic feature of metabolic syndrome [7]. Thus, DKD is the leading cause of ESRD and is associated with higher mortality rates, primarily due to cardiovascular complications arising from diabetes [70]. Nevertheless, future guidelines should be developed to address the treatment of hypertension in each type of diabetes mellitus.

DKD and Liver

Bile acids (BAs) are essential signaling molecules and co-metabolites involved in host–microbial interactions, playing a key role in regulating glycolipid metabolism. They have been shown to improve the prognosis of DKD by enhancing glucose and lipid metabolism, boosting energy metabolism, and stimulating renal BA receptors [4]. Future studies should explore the connection between DKD and bile acids in greater depth.

DKD and Bones

Osteocalcin (OCN) plays a crucial role in glucose metabolism. Numerous experimental studies have shown that OCN is involved in various stages of diabetes development, offering protection by influencing adipose tissue metabolism, pancreatic function, and oxidative stress. Sclerostin promotes adipogenesis and, given that adipose tissue has endocrine functions affecting energy metabolism, the endocrine role of bone is critical in diabetes research. Bone-derived hormones are linked to insulin secretion, insulin resistance, and glucose metabolism, making them promising therapeutic targets for diabetes and its complications due to their potential effectiveness in maintaining glucose homeostasis and bone health [51].

## 5. Limitations

Although the literature on DKD has progressed significantly, several issues and challenges remain, requiring further analysis and research. Limitations encountered include the complex, interconnected mechanisms underlying the pathogenesis of this disease [7]. Furthermore, many of the biomarkers discussed lack sensitivity and specificity testing, making their relevance a subject that requires validation through further studies [19]. Existing treatments, such as blood pressure control and RAS inhibitors, only slow disease progression without halting it, underscoring the need for more effective therapeutic strategies [69]. Finally, a lack of long-term studies has been observed, limiting the overall understanding of DKD’s natural history and the long-term efficacy and safety of emerging treatment options.

## 6. Conclusions

In conclusion, DKD represents a significant complication of diabetes, characterized by complex pathophysiological mechanisms, including hyperglycemia-induced oxidative stress, inflammation, and hemodynamic changes. Understanding these mechanisms is crucial for developing targeted therapies that can reverse the progression of DKD. Biomarkers, such as albuminuria, serum creatinine, and newer markers, like urinary podocyte-derived exosomes, have shown promise in early diagnosis and monitoring disease progression. Current treatment strategies, including optimal glycemic control, antihypertensive medications, and novel agents like SGLT2 inhibitors and finerenon, highlight the importance of a diversified approach. While DKD remains a disease that places multiple burdens on patients and their families, ranging from financial costs and treatment expenses to the support needed to navigate the various stages of treatment, continued research into the underlying mechanisms and potential therapeutic targets is essential for improving outcomes. This will ultimately lead to better management strategies and enhanced quality of life for those affected.

## Figures and Tables

**Table 1:** Summary of the DKD biomarkers, their functions, and their roles in DKD progression.

Biomarker	Function	Role in DKD
Albuminuria and Proteinuria	Protein leakage due to glomerular damage	Commonly used for DKD diagnosis but influenced by other factors like exercise and infections Not specific; additional biomarkers needed for early detection
Creatinine and eGFR	Measures kidney function through creatinine levels and GFR estimation	Common markers of kidney function but not specific for DKD; eGFR doesn’t reflect tubular damage Insufficient for assessing DKD severity; need for tubular markers
Cystatin C	Cysteine protease, better GFR indicator than creatinine	More reliable for GFR estimation, especially in early stages; reflects tubular injury with uCysC levels Affected by inflammation and fat mass; better for early DKD diagnosis
Kidney Injury Molecule-1 (KIM-1)	Transmembrane glycoprotein expressed in proximal tubule cells	Sensitive to tubular damage, linked to DKD progression, even in early stages with normo-albuminuria uKIM-1 is more specific than albumin for early progression
Neutrophil Gelatinase-Associated Lipocalin (NGAL)	Protein released upon nephron injury	Early marker for nephron damage; predicts albuminuria before it appears Distinguishes diabetic from non-DKD
Interleukin-6 (IL-6)	Inflammatory cytokine regulating immune response	Elevated in DKD; increases early before albuminuria and progresses with the disease Useful for assessing glomerular damage and disease progression
Transforming Growth Factor β (TGF-β)	Growth factor involved in fibrosis and glomerular hypertrophy	Linked to fibrosis and ECM deposition in DKD; elevated in patients with macroalbuminuria. Biomarker for predicting disease progression
Advanced Glycation End Products (AGEs)	Accumulation of modified proteins due to oxidative stress	Correlates with renal damage and dysfunction in DKD; elevated in ESRD Pentosidine is an early biomarker for DKD and linked to other complications like retinopathy
Monocyte Chemoattractant Protein 1 (MCP-1)	Inflammatory chemokine promoting renal inflammation	Elevated in urinary samples correlates with fibrosis and kidney damage Early nephropathy marker but doesn’t always predict albuminuria progression
Tumor Necrosis Factor α (TNF-α)	Pro-inflammatory cytokine interacting with TNF receptors	Contributes to kidney damage through inflammation; elevated in advanced stages of DKD Urinary TNF-α and circulating TNFR may serve as biomarkers for DKD progression
MicroRNAs	Small RNA sequences regulating gene expression	Altered in DKD, contributing to inflammation and kidney injury; certain miRNAs correlate with albuminuria Can serve as early biomarkers, influencing glomerular damage and fibrosis
Circular RNAs (circRNAs)	Stable RNA molecules influencing gene expression	Linked to gene regulation, inflammation, oxidative stress, and fibrosis in DKD. Potential biomarkers for monitoring disease progression in DKD
Fibroblast Growth Factor-23 (FGF-23)	Regulates phosphate levels, expressed by osteocytes and osteoblasts	Elevated in DKD, especially in early stages before phosphate levels rise; associated with inflammation. Useful as an early biomarker for DKD detection and monitoring
Vascular Endothelial Growth Factor (VEGF)	Growth factor regulating angiogenesis and kidney development	Early increase in expression linked to inflammation and hypoxia; contributes to glomerular damage and fibrosis. Dual role in repair and fibrosis; monitoring VEGF levels helps track disease progression
Connective Tissue Growth Factor (CTGF)	Mediates fibrosis and collagen expression	Elevated in DKD, correlates with proteinuria, serum creatinine, and fibrosis; independent predictor of ESRD Potential biomarker for monitoring DN progression
Hepcidin	Regulates iron hemeostasis by inhibiting iron absorption in the gut and its release from macrophages and liver stores	Predictive of clinical outcomes progression in DKD and its mortality
Urinary exosomes	Intercellular cargo transport, function depends on cargo content which includes proteins and RNA	Proteins or RNA contained in exosomes may be either increased or decreased depending on their roles. Certain exosomal marker levels may be altered early in the disease, serving as useful biomarkers for early kidney damage
Soluble Klotho	Product of cleavage of transmembrane α-Klotho protein, kidney is a major contributor to its levels. Implicated in aging through its role in phosphate homeostasis, integrity of blood vessels, and insulin signaling	Early decrease in plasma and urine levels in DN, progressive decrease in levels correlates with DKD progression Useful biomarker to monitor disease progression, however levels may be exaggerated as a result of aging
Osteopontin	Major sialoprotein in bone mineralization, remodeling, cellular adhesions, and various functions in various other cells, such as epithelial, endothelial, and renal cells	Alters podocyte motility and signaling which contributes to microalbuminuria, promotes interstitial macrophage buildup leading to tubulointerstitial damage Plasma levels correlated with eGFR Levels higher in DN, may serve as a biomarker to predict microalbuminuria early on
suPAR	Soluble form of urokine (a serine protease) receptor	Associated with increasing proteinuria, decreasing GFR in DM patients Biomarker for predicting kidney damage, potential for serum suPAR to be used for treatment follow-up
ß2 microglobulin	Part of MHC1 class, found on nucleated cells	Increased expression in urinary sediment in developing or established DN
